# Development and Validation of the Social–Emotional Competence Questionnaire for College Students

**DOI:** 10.3390/bs16061024

**Published:** 2026-06-18

**Authors:** Chao Li, Xiuli Liu

**Affiliations:** 1 Faculty of Education, Northeast Normal University, Changchun 130024, China; lichao167@nenu.edu.cn; 2 Student Psychological Development and Guidance Center, Beihua University, Jilin 132013, China; 3 School of Psychology, Northeast Normal University, Changchun 130024, China

**Keywords:** college students, Social–Emotional competence, scale development, reliability, validity

## Abstract

This study aimed to develop and validate a Social–Emotional Competence instrument for college students. The questionnaire includes 30 items across five dimensions: self-awareness, self-management, social awareness, interpersonal communication, and sense of responsibility. The items were selected from an initial pool of 42 items generated through a comprehensive literature review, semi-structured interviews, and expert evaluation. A total of 1008 valid responses were collected from undergraduate students. The dataset was randomly divided into two independent samples. Sample 1 (*n* = 504) was used for item analysis and exploratory factor analysis, while Sample 2 (*n* = 504) was employed for confirmatory factor analysis (CFA) and assessing the reliability and validity of the questionnaire. Exploratory factor analysis supported a five-factor structure, accounting for 60.619% of the total variance. Confirmatory factor analysis indicated that the model fit the data reasonably well, with CFI = 0.915, TLI = 0.905, RMSEA = 0.063, and SRMR = 0.046. The questionnaire demonstrated excellent internal consistency (α = 0.958) and maintained strong stability over time, as evidenced by a test–retest correlation of r = 0.939. Criterion-related validity was supported by significant positive correlations with interpersonal competence and negative correlations with emotion regulation difficulties and depressive symptoms. Taken together, these results provide preliminary support for the reliability and validity of the instrument, suggesting that it may serve as a practical tool for evaluating social–emotional competence among college students.

## 1. Introduction

Social and emotional competence (SEC) refers to an integrated set of abilities that enable individuals to effectively regulate their emotions and behavior, respect others, establish positive interpersonal relationships, and make responsible decisions (Corcoran et al., 2020; Napolitano et al., 2021; Liu et al., 2023). In recent years, SEC has attracted increasing attention from scholars. A substantial body of research has demonstrated that SEC is a core psychological trait for promoting holistic development and is closely associated with academic engagement, academic performance, mental health, and adaptive functioning (Domitrovich et al., 2017; Li et al., 2024; Durlak et al., 2011; Ghasemi et al., 2023; Nicoll, 2014).

Measurement of SEC has primarily been guided by three distinct models. The five-dimensional framework proposed by the Collaborative for Academic, Social, and Emotional Learning categorizes SEC into self-awareness, self-management, social awareness, relationship skills, and responsible decision-making (CASEL, 2017). Based on this framework, the Delaware Social–Emotional Competency Scale (DSECS-S) has been widely applied. However, the DSECS-S includes only four dimensions—self-management, social awareness, relationship skills, and responsible decision-making—omitting the core construct of self-awareness (Mantz et al., 2018). Self-awareness plays a crucial role in individual development, particularly for college students in emerging adulthood.

The Organization for Economic Co-operation and Development (OECD) developed a measurement framework based on Big Five personality theory and ecological systems theory. This framework offers a structured approach to examining how different social and emotional skills function across educational and life contexts. For instance, openness relates to creativity, curiosity, and adaptability; conscientiousness relates to self-discipline, persistence, organizational skills, and time management; extraversion relates to social skills, communication, and teamwork; agreeableness relates to empathy, cooperation, and conflict resolution; and neuroticism relates to emotion regulation, stress resilience, and self-confidence. Each Big Five dimension is further linked to specific social and emotional skills. While this framework is particularly strong in assessing personality traits, it is less effective in capturing the developmental potential of SEC (Chernyshenko et al., 2018).

The Ministry of Education of the People’s Republic of China, in collaboration with UNICEF, proposed the “Children’s Social and Emotional Learning Framework” in 2018, based on cooperative Social–Emotional learning programs. This framework specifies SEC using a three-by-six-factor model encompassing six core capacities: self-awareness, self-management, awareness of others, management of others, collective awareness, and collective management (Chen & Yu, 2022). Although the framework is highly localized, it emphasizes relational aspects while paying relatively less attention to emotional processes. Furthermore, it was primarily designed for primary and secondary education and does not fully address the psychological aspects of college students.

Existing instruments for assessing social–emotional competence in college students have several limitations. Some do not cover key dimensions, such as self-awareness, while others rely mainly on personality models rather than developmental frameworks. In addition, some tools were originally developed for younger populations and may not fully reflect the psychological characteristics and developmental needs of students in higher education. Overall, these limitations suggest that current measures may not provide a sufficiently comprehensive or developmentally suitable assessment of Social–Emotional competence among Chinese college students. Therefore, a new instrument based on an appropriate developmental framework is needed.

Among the theoretical models available, the CASEL framework is widely recognized for its balanced focus on emotional awareness, self-regulation, interpersonal functioning, and responsible decision-making. Compared with personality-based or age-specific models, it provides a more developmentally oriented and integrative perspective, making it particularly suitable for capturing the different aspects of social–emotional competence in higher education. This makes it an appropriate conceptual foundation for developing a new measurement tool.

Based on the CASEL framework, the study aimed to develop and validate a Social–Emotional Competence Questionnaire for Chinese college students. The study examined three primary objectives: first, to evaluate whether the questionnaire replicates the five-factor structure proposed by CASEL; second, to assess the internal consistency and structural validity of the subscales; and third, to examine the associations between questionnaire scores and established measures of social–emotional functioning, including interpersonal competence, emotion regulation difficulties, and depressive symptoms. Based on prior literature and theoretical considerations, it was hypothesized that the questionnaire would demonstrate a five-factor structure consistent with the CASEL framework, that theoretically related factors would show positive intercorrelations, and that total and subscale scores would be positively associated with interpersonal competence and negatively associated with difficulties in emotion regulation and depressive symptoms. These a priori expectations provide a clear conceptual and psychometric basis for subsequent exploratory and confirmatory analyses.

## 2. Methods

### 2.1. Questionnaire Development

Based on a comprehensive review of domestic and international SEC measurement tools and relevant theoretical frameworks, an initial item pool was constructed in accordance with established frameworks in the Social–Emotional learning field. Existing instruments were consulted, and items were drafted to reflect the daily experiences of college students and the specific objectives of the present study. This process resulted in an initial questionnaire comprising 45 items across five core dimensions: self-awareness, self-management, social awareness, interpersonal communication, and sense of responsibility.

To refine the items, semi-structured interviews were conducted with 21 undergraduate students (age range 17–22). Participants described their experiences and perceptions related to Social–Emotional competence in academic and social contexts. Based on thematic analysis of the interview data, minor wording adjustments were made to enhance clarity and relevance, and 3 items were added, resulting in a total of 48 items.

Subsequently, three experts in psychology, each with the rank of associate professor or higher, evaluated the content validity of the items. Their assessment focused on the clarity of item wording, alignment with the defined dimensions, and cultural appropriateness. Based on the experts’ feedback, 6 items were removed due to redundancy or low relevance, and several items were revised for improved clarity, resulting in the final 42-item questionnaire. Although the number of experts was limited and formal content validity indices (e.g., CVR or CVI) were not calculated, using expert review to guide item refinement is a commonly accepted practice in early-stage questionnaire development (Lynn, 1986; Polit & Beck, 2006).

The questionnaire employed a 5-point Likert scale (1 = strongly disagree, 2 = disagree, 3 = neither disagree nor agree, 4 = agree, 5 = strongly agree), with no reverse-scored items. All items were positively worded to maintain conceptual clarity and reduce potential confusion, although the possibility of acquiescence bias is acknowledged. Higher total scores indicate higher levels of social–emotional competence. The finalized questionnaire was then administered to a large sample of undergraduate students to evaluate its psychometric properties.

### 2.2. Participants

Using a cluster sampling approach, 1200 questionnaires (in Chinese) were distributed to undergraduate students at a university, yielding 1008 valid responses and an effective response rate of 84%. Among the participants, 346 (34.3%) were male, and 662 (65.7%) were female. The distribution by academic year was as follows: 423 (42.0%) first-year students, 214 (21.2%) second-year students, 286 (28.4%) third-year students, and 85 (8.4%) fourth-year students. Participants’ ages ranged from 17 to 25 years. The sample also included students from a variety of academic disciplines, including humanities (*n* = 299), natural sciences (*n* = 277), engineering (*n* = 194), medical sciences (*n* = 145), and arts and physical education (*n* = 93). To examine the structural validity and reliability of the questionnaire, the total sample was randomly divided into two independent subsamples. Sample 1 (*n* = 504) was used for item analysis and exploratory factor analysis (EFA) to identify the underlying factor structure. Sample 2 (*n*= 504) was used for confirmatory factor analysis (CFA) to verify the factor structure identified in the EFA, as well as for internal consistency reliability analysis and criterion-related validity assessment through correlations with external measures. From Sample 2, a subset of 120 students was randomly selected for a test–retest procedure, resulting in 104 valid responses (Sample 3), with a retest interval of four weeks.

### 2.3. Criterion Measures

To assess the validity of the SEC Questionnaire for college students, three widely used instruments with established reliability and validity in Chinese populations were selected as criterion measures: the short form of the Interpersonal Competence Questionnaire (ICQ-15), the Difficulties in Emotion Regulation Scale (DERS-16), and the Beck Depression Inventory (BDI). These measures were selected because they represent theoretically distinct yet related aspects of socio-emotional functioning relevant to the SEC construct. The ICQ-15 was used to assess interpersonal competence as a positive indicator of social functioning; the DERS-16 was used to measure difficulties in emotion regulation as a theoretically related construct reflecting self-regulatory processes; and the BDI was included as a negative psychological outcome reflecting depressive symptoms. These instruments are closely related to the relevant dimensions of Social–Emotional competence and provide evidence for the construct validity of the SEC scale through criterion-related associations.

Interpersonal Competence Questionnaire—Short Form (ICQ-15): The ICQ-15, developed by Coroiu et al. (2015) based on the original 40-item ICQ by Buhrmester et al. (1988), comprises 15 items across five dimensions: initiating relationships, expressing refusal, self-disclosure, providing emotional support, and conflict management (three items per dimension). Responses are rated on a 5-point Likert scale (1 = very difficult, 5 = very skilled), with higher scores indicating greater interpersonal competence. In the present study, the ICQ-15 demonstrated excellent internal consistency, with a Cronbach’s α of 0.937.

Difficulties in Emotion Regulation Scale—Short Form (DERS-16): Developed by Bjureberg, Gratz, and colleagues based on the original 36-item DERS, the DERS-16 contains 16 items assessing multidimensional difficulties in regulating negative emotions. It includes five dimensions: nonacceptance of emotional responses, difficulties engaging in goal-directed behavior, impulse control difficulties, limited access to emotion regulation strategies, and lack of emotional clarity. Items are rated on a 5-point Likert scale, with higher scores indicating greater emotion regulation difficulties (Bjureberg et al., 2016). In the present study, the DERS-16 demonstrated excellent internal consistency (Cronbach’s α = 0.965).

Beck Depression Inventory—Short Form (BDI-13): The BDI-13, developed by Aaron T. Beck and colleagues as a shortened version of the original Beck Depression Inventory, is a classic instrument for assessing depressive symptoms. It includes 13 items covering emotional, behavioral, cognitive, and physiological aspects of depression. Each item reflects a specific depressive symptom and is rated on a 4-point Likert scale (0–3), indicating severity from mild to severe (Beck & Beck, 1972). In the present study, the BDI demonstrated good internal consistency (Cronbach’s α = 0.892).

### 2.4. Statistical Analysis

Data analysis was conducted using SPSS (version 25.0) for item analysis, exploratory factor analysis, correlation analysis, and internal consistency reliability analysis, while AMOS 26.0 was utilized for confirmatory factor analysis.

## 3. Results

### 3.1. Item Analysis

Total scores for the 42 items were calculated by summing individual item responses. For Sample 1 (*n* = 504), participants were ranked from highest to lowest total score. The top 27% and bottom 27% of participants were designated as the high-score and low-score groups, with total scores of 180 and 160, respectively. Independent-samples *t*-tests were conducted to compare the mean scores of each item between these two groups. The results indicated that the mean scores of all 42 items differed significantly (*p* < 0.001 for all items), demonstrating that all items had adequate discriminative power.

### 3.2. Exploratory Factor Analysis

Exploratory factor analysis (EFA) was performed on the initial 42-item questionnaire using data from Sample 1 *n* = 504). The Kaiser–Meyer–Olkin (KMO) measure of sampling adequacy was 0.961, and Bartlett’s test of sphericity was statistically significant (χ^2^ = 13,534.687, df = 861, *p* < 0.001), indicating that the data were appropriate for factor analysis. Principal component analysis (PCA) was employed as an initial exploratory procedure for item reduction and to identify the underlying structure prior to confirmatory factor analysis. Although common factor analysis is often preferred for latent construct development, PCA was used here as an initial, data-driven procedure. This approach helped reduce the number of items and improve factor interpretability, consistent with early-stage scale development practices in the social sciences (Fabrigar et al., 1999). Varimax rotation was applied to enhance the interpretability of the factor solution. Although varimax rotation assumes orthogonal factors, the subsequent CFA tested the latent factor structure and accounted for correlations among constructs, providing a more accurate representation of the underlying dimensions. During the factor extraction process, items were removed sequentially based on the following criteria: (1) absolute factor loadings below 0.45; (2) communalities below 0.40; (3) cross-loadings above 0.40 on two or more factors; and (4) factors containing fewer than three items. Three rounds of item deletion were performed to refine the questionnaire and improve factor interpretability. In the first round, 10 items were removed. Of these, 3 were due to low factor loadings (<0.45), 6 were due to high cross-loadings (>0.40) on multiple factors, and 1 was removed because the factor contained fewer than three items. In the second round, 2 additional items were deleted: 1 due to low communalities and factor loading, and 1 due to cross-loadings. No items were removed in the third round. Following this iterative process, a final set of 30 items was retained for subsequent factor analysis. Detailed information on the item deletion process is provided in Table 1.

EFA was then conducted on these 30 retained items. The analysis suggested a five-factor solution, with all retained factors having eigenvalues greater than 1. Following varimax rotation, the eigenvalues of these factors ranged from 2.218 to 5.305. Together, the five-factor structure accounted for 60.619% of the total variance. The standardized factor loadings of the items on their respective factors are presented in Table 2.

Based on theoretical considerations and item content, Factors 1 through 5 were labeled as Self-Management, Sense of Responsibility, Social Awareness, Interpersonal Communication, and Self-Awareness, respectively.

Self-Management comprises nine items (Items 4, 5, 6, 7, 8, 9, 10, 11, 12) and reflects an individual’s intrinsic motivation when facing challenges. This includes self-encouragement, maintaining optimism, persistence toward goals, coping with stress, and learning from experiences.

Sense of Responsibility consists of six items (Items 25, 26, 27, 28, 29, 30) and represents an individual’s sense of responsibility toward oneself, others, and the collective. This dimension encompasses making responsible decisions, considering the consequences of actions, adhering to social norms, and demonstrating prudence in task management.

Social Awareness includes seven items (Items 18, 19, 20, 21, 22, 23, 24) and captures an individual’s understanding of and concern for others’ emotions. It involves empathizing with others’ experiences, attending to others’ feelings, maintaining positive interpersonal relationships, and perceiving the emotional states of others.

Interpersonal Communication comprises five items (Items 13, 14, 15, 16, 17) and reflects an individual’s communication and conflict-resolution skills in social interactions. This dimension includes initiating conversations with strangers, expressing oneself clearly, resolving misunderstandings through communication, managing interpersonal conflicts, and forming friendships with people from diverse backgrounds.

Self-Awareness consists of three items (Items 1, 2, 3) and represents an individual’s recognition of their own emotions, attitudes, and values. This includes identifying situations that trigger emotions, acknowledging the significance of one’s personal viewpoints, and understanding one’s strengths and weaknesses.

### 3.3. Validity Analysis

#### 3.3.1. Construct Validity

Confirmatory factor analysis (CFA) was conducted on the 30-item questionnaire using data from Sample 2 (*n* = 504). The hypothesized model was the five-factor structure derived from the previous exploratory factor analysis. To determine whether the five-factor model provided the best fit, it was compared with four competing models:

One-factor model: All five factors were combined into a single factor.

Three-factor model: Social Awareness and Sense of Responsibility were combined into one factor, while Self-Awareness and Self-Management were merged into another factor, reflecting their strong associations and overlap in practical applications.

Four-factor model: Social Awareness and Sense of Responsibility were combined into one factor, based on their theoretical relatedness and correlations observed in the exploratory factor analysis.

Five-factor model with added residual covariances: The original five-factor model was further modified by including residual covariance terms between selected items to improve model fit.

**Figure 1:**
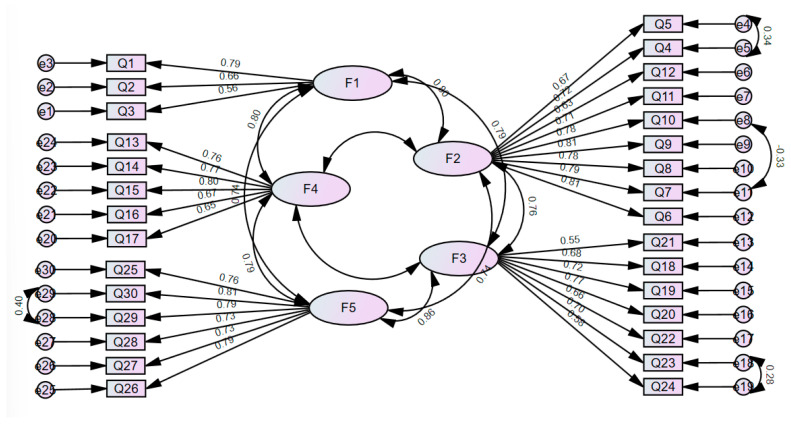
Confirmatory factor analysis model of the Social–Emotional Competence Questionnaire for College Students.

Table 3 presents the fit indices for the CFA models. The original five-factor model demonstrated an acceptable fit; however, modification indices suggested that four pairs of items had correlated residuals (Items 4 and 5; Items 7 and 10; Items 23 and 24; Items 29 and 30). Based on theoretical justification, residual covariances were added for these four pairs, resulting in a modified five-factor model with substantially improved fit indices. The final model is depicted in Figure 1. These findings indicate that the modified model provides a better representation of the underlying factor structure and exhibits strong construct validity.

#### 3.3.2. Convergent and Discriminant Validity

To examine the construct validity of the Social–Emotional Competence Questionnaire, composite reliability (CR), average variance extracted (AVE), and the square roots of AVE were calculated for each subscale. As shown in Table 4, all CR values exceeded 0.70, indicating good internal consistency. AVE values ranged from 0.448 to 0.589; although two subscales had AVE slightly below the commonly recommended threshold of 0.50, the values were considered acceptable given the high CR. The square roots of AVE ranged from 0.669 to 0.768, which were greater than the correlations between the corresponding constructs, providing additional support for convergent validity.

Discriminant validity was further assessed using the Heterotrait–Monotrait ratio (HTMT). All HTMT values ranged from 0.669 to 0.818, below the recommended threshold of 0.85, indicating adequate discriminant validity.

#### 3.3.3. Criterion-Related Validity

As shown in Table 5, the total score of the SEC Questionnaire was significantly positively correlated with the total score of the Interpersonal Competence Questionnaire (r = 0.73, *p* < 0.01). Moreover, all five subscale scores of the Social–Emotional Competence Questionnaire were significantly positively correlated with the total score of the Interpersonal Competence Questionnaire.

Conversely, the total score of the Social–Emotional Competence Questionnaire was significantly negatively correlated with the total score of the Difficulties in Emotion Regulation Scale (r = −0.41, *p* < 0.01), and all subscale scores were likewise significantly negatively correlated with the total score of the DERS (see Table 6). Similarly, the total score of the Social–Emotional Competence Questionnaire was significantly negatively correlated with the total score of the Beck Depression Inventory (r = −0.49, *p* < 0.01), with all subscale scores showing significant negative correlations as well (see Table 7).

In summary, these results indicate that both the total score and the subscale scores of the Social–Emotional Competence Questionnaire are positively associated with interpersonal competence and negatively associated with difficulties in emotion regulation and depressive symptoms, consistent with theoretical expectations. College students with higher levels of social–emotional competence generally demonstrate stronger interpersonal communication, fewer emotion regulation difficulties, and lower levels of depression.

The Social–Emotional Competence Questionnaire developed in this study effectively captures individuals’ functioning across key domains, including social interaction, emotion regulation, and psychological well-being, demonstrating good criterion-related validity. These findings further support the theoretical assertion that social–emotional competence constitutes a foundational ability for college students’ adaptation and mental health, and they confirm the utility of this instrument in assessing student development and psychological well-being in higher education contexts.

### 3.4. Reliability Analysis

Internal consistency of the questionnaire was assessed using data from Sample 2. The Social–Emotional Competence Questionnaire demonstrated excellent internal consistency, with a Cronbach’s α of 0.958 for the overall scale. The Cronbach’s α coefficients for the subscales were as follows: Self-Awareness, 0.779; Self-Management, 0.915; Social Awareness, 0.848; Interpersonal Communication, 0.845; and Sense of Responsibility, 0.897.

Test–retest reliability was examined using Sample 3, in which 104 participants completed the questionnaire again after a four-week interval. Pearson correlation analyses indicated a test–retest reliability coefficient of 0.939 for the overall scale. Subscale coefficients were 0.706 for Self-Awareness, 0.920 for Self-Management, 0.863 for Social Awareness, 0.809 for Interpersonal Communication, and 0.880 for Sense of Responsibility. All subscale test–retest coefficients ranged from 0.706 to 0.920, exceeding the generally accepted threshold of 0.70.

## 4. Discussion

### 4.1. Reliability and Validity of the Social–Emotional Competence Questionnaire for Chinese College Students

Grounded in theoretical frameworks of social–emotional competence and the developmental context of Chinese college students, this study developed the Social–Emotional Competence Questionnaire and evaluated its psychometric properties. These results provide preliminary evidence supporting the reliability and validity of the questionnaire. Overall, the findings indicate that the questionnaire meets acceptable standards for construct validity, internal consistency, test–retest reliability, and criterion-related validity, providing preliminary support for its scientific rigor and reliability as a tool for measuring Social–Emotional competence among students at the sampled university or in similar institutional contexts.

Regarding construct validity, exploratory factor analysis extracted five factors that collectively accounted for 60.619% of the total variance, indicating that the questionnaire captures the major dimensions of social–emotional competence, consistent with the theoretical framework proposed by CASEL (2017). This amount of explained variance is consistent with common benchmarks reported in social science questionnaire development, suggesting that the identified factors meaningfully represent underlying psychological constructs. Confirmatory factor analysis further supported the five-factor structure, with fit indices indicating an acceptable model fit (CFI = 0.915, TLI = 0.905, RMSEA = 0.063, SRMR = 0.0464), providing evidence consistent with theoretically meaningful dimensions of social–emotional competence. It should be noted that certain items retained under the Social Awareness factor, including Item 19 (“I am able to maintain good relationships with my family and friends”), Item 23 (“I receive substantial support from close family members and friends”), and Item 24 (“I have one or more close friends”), could be interpreted as reflecting social outcomes or resources rather than awareness per se. However, consistent with the CASEL (2017) framework and empirical research on social awareness (Durlak et al., 2011; Zins et al., 2004), these items are conceptually aligned with Social Awareness in the Chinese university context. In collectivist cultures, social awareness is closely tied to perceptions of social support, connectedness, and the maintenance of positive relationships, making these items culturally relevant indicators of the construct. The distinction between competence, social functioning, and social resources should be made clearer. Our items are designed to capture social awareness in real-world contexts, not as a purely abstract ability. This classification is based on the Chinese cultural context and may change with future research. More work is needed to test the boundaries between these constructs and, if necessary, refine the items. However, these results should be interpreted cautiously, as they are based on self-report data and a single-sample design, which may introduce shared method variance.

In this study, exploratory factor analysis (EFA) was performed using principal component analysis (PCA) with varimax rotation. This approach helped to reduce the initial item pool and clarify the factor structure. However, it should be noted that PCA primarily captures total variance rather than shared variance among items, and varimax rotation assumes orthogonal factors. As Social–Emotional competence dimensions are expected to be correlated, this method may not fully reflect the underlying relationships. To address this issue, we conducted confirmatory factor analysis (CFA) using a correlated factor model, which better accounts for the expected interrelationships among dimensions. Nevertheless, the results from the exploratory phase should be interpreted with caution. Future studies could consider using oblique rotations or alternative common-factor analytic techniques to further examine the latent structure and enhance the robustness of the scale.

Furthermore, a few residual covariances were allowed between items within the same dimension (Items 4 and 5, 7 and 10, 23 and 24, 29 and 30) to account for their conceptual overlap. These modifications were theoretically justified because the items are conceptually related, capturing closely associated aspects of the same construct. Allowing such residual correlations is a common practice in CFA to account for shared method variance and improve model fit without compromising the underlying factor structure. It should also be noted that the allowed residual covariances between certain paired items do not indicate item redundancy but rather reflect theoretically expected conceptual overlap within each construct. In the context of Chinese university students, such overlaps are culturally consistent, as multiple items may capture different aspects of a construct that are closely interconnected in everyday social–emotional functioning. Therefore, the residual correlations are theoretically justified, rather than purely data-driven, and support the validity of the five-factor structure.

Although the GFI and AGFI values were slightly below 0.90, such results are common in CFA models with multiple items and complex structures. Previous studies have shown that these indices are sensitive to item quantity and model complexity and may underestimate model fit in multi-factor, item-level analyses (Marsh et al., 2004). Therefore, evaluating model fit should rely on a combination of indices that are more robust to complexity, such as CFI, TLI, RMSEA, and SRMR. Based on this overall pattern, the five-factor structure proposed in this study can be regarded as demonstrating good construct validity.

From a theoretical standpoint, the CASEL framework explicitly defines five core dimensions of social–emotional competence: Self-Awareness, Self-Management, Social Awareness, Interpersonal Communication, and Responsible Decision-Making (CASEL, 2017). However, some widely used instruments do not fully capture this five-dimensional structure, with the Self-Awareness dimension often underrepresented (Mantz et al., 2018). It is frequently integrated into constructs such as Self-Management or Emotion Regulation, partially weakening the theoretical model’s complete representation at the measurement level.

For college students in emerging adulthood, self-awareness is particularly critical for the formation of values, role identity, and emotion regulation, and its developmental level directly influences social adaptation and psychological well-being (Arnett, 2000). Consequently, systematically including Self-Awareness in measures of Social–Emotional competence holds important theoretical and practical significance.

The questionnaire demonstrated high overall internal consistency, with a Cronbach’s α of 0.958, suggesting that the items are largely consistent in measuring the intended construct. Although this high α supports reliability, it may also suggest a certain degree of item redundancy, indicating that some items could be highly correlated or overlapping in content (Clark & Watson, 1995).

Internal consistency for the subscales ranged from acceptable to excellent (α = 0.779–0.915). The Self-Awareness subscale, with an α of 0.779, was slightly lower than the other subscales, warranting further consideration. The Self-Awareness construct encompasses individuals’ awareness of their emotions, strengths, values, and behavioral motivations. This is a relatively complex psychological function influenced by multiple factors, including metacognitive ability, reflective habits, and emotional awareness. Such complexity may reduce inter-item consistency relative to other behavioral or emotional dimensions. Additionally, this subscale contains fewer items, and Cronbach’s α is known to be sensitive to item number, with fewer items tending to yield lower α values. Overall, although slightly lower than other subscales, the internal consistency of Self-Awareness remains within an acceptable range. It should be noted that one item in the Self-Awareness subscale, “I believe that my opinions are worthy of being heard by others,” could be interpreted as reflecting self-esteem, assertiveness, or self-worth rather than purely self-awareness. However, in the context of Chinese college students, this item is conceptually aligned with self-awareness, as recognizing the value of one’s own opinions reflects metacognitive awareness and self-reflection—key components of the construct. Future studies may consider adding additional items to more fully capture the multidimensional nature of self-awareness and to enhance the internal consistency of the subscale.

Test–retest reliability was examined using a subset of participants who completed the questionnaire again after a four-week interval, yielding a coefficient of 0.939. This indicates good temporal stability, consistent with social–emotional competence being a relatively stable psychological trait. High test–retest reliability also suggests that the questionnaire is resistant to short-term fluctuations caused by situational factors or transient emotional states, supporting its utility for research and practice.

The total score of social–emotional competence was significantly positively correlated with interpersonal competence, with a relatively high correlation coefficient, indicating a close relationship between social–emotional competence and actual social functioning. This supports the criterion-related validity of the questionnaire for assessing social functioning and aligns with emotional intelligence theory: individuals with higher emotional awareness, listening skills, empathy, and communication ability are more likely to receive positive feedback in social interactions and establish stable interpersonal networks (Mayer et al., 2008).

Social–emotional competence was negatively correlated with difficulties in emotion regulation, consistent with emotion regulation theory. Components such as self-awareness, self-management, and the ability to recognize others’ emotions directly influence the choice and effectiveness of emotion regulation strategies (Denham & Brown, 2010). Consequently, college students with higher social–emotional competence generally employ more effective emotion regulation strategies, reducing emotional dysregulation and difficulties in emotional expression. These findings highlight social–emotional competence as a foundational ability for emotional functioning and support the questionnaire’s utility in identifying emotion-related difficulties and assessing emotion regulation skills.

Furthermore, social–emotional competence was negatively correlated with depressive symptoms, suggesting a protective effect on college students’ mental health. Previous research indicates that well-developed emotional awareness and regulation skills help individuals cope effectively with negative emotions, thereby reducing the risk of depressive symptoms (Aldao et al., 2010). Stable and supportive interpersonal networks, as reflected in perceived social support, act as a psychological resource that predicts lower levels of depression among college students (Tian et al., 2025). The present findings are highly consistent with these prior studies, further supporting the notion that Social–Emotional competence is a key ability related to mental health and suggesting that the questionnaire may be useful for future research examining mental health outcomes.

During the evaluation of convergent and discriminant validity, additional psychometric evidence was examined. Specifically, two subscales—Self-Awareness and Social Awareness—showed AVE values slightly below the conventional threshold of 0.50 (Fornell & Larcker, 1981; Hair et al., 2019), whereas CR values for all subscales exceeded 0.70. According to established psychometric guidelines, an AVE below 0.5 is permissible when CR is adequate, particularly for subscales with a limited number of items or measuring complex psychological constructs (Fornell & Larcker, 1981). These findings suggest that, despite slightly lower AVE, the subscales reliably reflect the intended constructs and maintain acceptable convergent validity.

Discriminant validity was further assessed using the Heterotrait–Monotrait ratio (HTMT). Most subscale pairs demonstrated HTMT values below 0.80, indicating a clear distinction among the constructs. Two pairs—Self-Management with Interpersonal communication and Social Awareness with Interpersonal communication—exceeded 0.80 but remained below the conservative threshold of 0.85 suggested by Henseler et al. (2015) and Kline (2016). These moderately high values likely reflect conceptual relatedness between these constructs, as interpersonal communication inherently involves elements of self-management and social awareness. Nonetheless, each subscale retained sufficient distinctiveness, providing empirical support for discriminant validity. In summary, the Social–Emotional Competence Questionnaire demonstrated high internal consistency and strong temporal stability at both overall and subscale levels. The observed correlations with external criterion measures were consistent with theoretical expectations, indicating satisfactory construct, content, and criterion-related validity. These results suggest that the questionnaire provides a stable and reliable measure of Social–Emotional competence for research purposes, offering preliminary support for its use in similar university contexts.

### 4.2. Theoretical and Practical Implications

From a theoretical standpoint, the Social–Emotional Competence Questionnaire developed in this study aligns with the international SEL framework while incorporating the learning context and developmental characteristics of Chinese college students. Core dimensions, such as Self-Awareness, are retained and emphasized. The questionnaire demonstrated satisfactory construct and criterion-related validity, providing a standardized measurement tool for subsequent research on Social–Emotional competence. It contributes to the accumulation of localized evidence regarding the structure of Social–Emotional competence among Chinese college students and offers a reliable measurement foundation for future SEL-related intervention studies.

From a practical perspective, the questionnaire provides a reliable tool to assess college students’ social–emotional competence across five dimensions: Self-Awareness, Self-Management, Social Awareness, Interpersonal Communication, and Sense of Responsibility. The instrument can help universities identify students’ relative strengths and areas for development. While this study does not evaluate predictive or intervention outcomes, the questionnaire offers a foundation for future research and practice aimed at monitoring students’ social–emotional functioning and informing the design of targeted programs.

### 4.3. Limitations and Future Directions

Despite achieving satisfactory reliability and validity, several limitations of the present study should be acknowledged. First, the sample was drawn from a single university, and although students from multiple academic disciplines were included, the generalizability of the findings remains limited to similar institutional contexts. Future research should recruit participants from diverse regions and university types to enhance the generalizability of the findings. Second, the Self-Awareness subscale contained relatively few items, which may have contributed to its lower internal consistency. Future studies could improve the instrument’s effectiveness by increasing the number of items or conducting in-depth interviews to refine the measurement. Third, this study primarily relied on self-report measures, which are susceptible to social desirability bias. Subsequent research could incorporate alternative assessment methods—such as behavioral observation, peer evaluation, or interview data—to further enhance the reliability and validity of the questionnaire and provide a more comprehensive assessment of college students’ Social–Emotional competence. Additionally, all items in the questionnaire were positively worded to maintain conceptual clarity and reduce potential confusion among respondents. However, this approach may increase susceptibility to acquiescence bias, which could affect participants’ responses. Future studies could consider incorporating reverse-scored items or other strategies to mitigate this potential bias. Finally, although the modified CFA model demonstrated acceptable fit, it was optimized within the same sample, and no independent cross-validation was conducted. Therefore, the proposed factor structure requires confirmation in future studies using independent samples. In addition, the current study relied on cross-sectional data, which is common in scale development but limits the ability to evaluate temporal stability and predictive validity. Future research should include longitudinal designs to examine measurement invariance across time and predictive relationships, thereby providing more robust evidence for the factor structure and overall validity of the questionnaire.

## 5. Conclusions

In conclusion, this study developed and validated the Social–Emotional Competence Questionnaire for Chinese college students. The instrument demonstrated high reliability, including strong internal consistency and robust test–retest stability, as well as satisfactory construct, convergent, and discriminant validity. Both exploratory and confirmatory factor analyses supported a five-factor structure consistent with established theoretical frameworks of Social–Emotional competence. The questionnaire provides a scientifically grounded tool for assessing students’ strengths and developmental needs across five domains: Self-Awareness, Self-Management, Social Awareness, Interpersonal Communication, and Sense of Responsibility (See Appendix A). These findings contribute to the localized evidence base for measuring social–emotional competence in Chinese higher education settings. Future studies could further evaluate the instrument’s longitudinal stability, predictive validity, and applicability across diverse cultural contexts. Additionally, the questionnaire may serve as a practical foundation for designing targeted interventions and monitoring students’ Social–Emotional development over time.

## Figures and Tables

**Table 1:** Sequential item reduction process and rationale in exploratory factor analysis.

Deletion Round	Item Content	Reason for Deletion
Round 1 Round 2	I am able to recognize and respect others’ opinions and feelings. I can get along well with others. I can understand others’ thoughts through their behavior. Even when facing difficulties, I do not blame external circumstances. After disagreements with family or friends, I am willing to initiate communication. In a team, I can cooperate effectively with others and complete assigned tasks. In my view, greeting others proactively is an important part of social interaction. I can understand others even when their views differ from mine. I feel that I deserve respect from others. I know that others’ praise is important to me. Generally speaking, I can forgive others’ mistakes. I carefully complete the tasks assigned to me.	Low factor loading (<0.45) Low factor loading (<0.45) Low factor loading (<0.45) High cross-loading (>0.40) High cross-loading (>0.40) High cross-loading (>0.40) High cross-loading (>0.40) High cross-loading (>0.40) High cross-loading (>0.40) Fewer than three items in a factor Low communality (<0.40) Low factor loading (<0.45) High cross-loading (>0.40)

Note. The item texts presented in this table were translated from the original Chinese version.

**Table 2:** Results of the exploratory factor analysis (N = 504).

Items	Factor Loadings				
	1	2	3	4	5
Q8. I believe that what others can accomplish, I can also achieve through effort.	0.744				
Q11. When facing difficulties, I encourage and motivate myself.	0.712				
Q6. When encountering difficulties, I am confident in my ability to overcome them.	0.710				
Q9. I firmly believe that I can achieve the goals I set for myself.	0.708				
Q7. I am able to maintain an optimistic mindset over time.	0.708				
Q12. I believe that once something is planned, it should be followed through to completion.	0.695				
Q4. I can effectively alleviate my own stress.	0.651				
Q10. I am able to learn from my successes and accumulate experience.	0.616				
Q5. I am able to regulate my behavior.	0.584				
Q29. The decisions I make are not only responsible to myself but also to others and the collective.		0.763			
Q30. I am able to weigh the impact of my decisions on myself, others, and the collective, and make appropriate adjustments when necessary.		0.746			
Q26. I am able to take responsibility for my actions.		0.743			
Q25. When making choices or decisions, I consider social safety and moral norms.		0.713			
Q27. I am able to correctly distinguish right from wrong.		0.676			
Q28. In dealing with people and situations, I think carefully before acting.		0.528			
Q22. When I see others experiencing misfortune, I feel deep sympathy.			0.683		
Q20. I care about others’ feelings.			0.659		
Q19. I am able to maintain good relationships with my family and friends.			0.615		
Q23. I receive substantial support from close family members and friends.			0.594		
Q21. I am able to sense when others are unhappy.			0.584		
Q18. I am able to accept people with different personality traits.			0.508		
Q24. I have one or more close friends.			0.482		
Q16. I am able to initiate conversations with strangers easily.				0.677	
Q15. When communicating with others, I can clearly express my thoughts and feelings.				0.670	
Q14. I am able to resolve misunderstandings with others through communication.				0.652	
Q13. I am skilled at handling conflicts with others.				0.645	
Q17. I am able to make friends with different types of people.				0.574	
Q2. I know under what circumstances I tend to become angry.					0.727
Q3. I believe that my opinions are worthy of being heard by others.					0.710
Q1. I have a clear understanding of my strengths and weaknesses.					0.684
Eigenvalue	5.305	4.027	3.370	3.265	2.218
Variance Contribution Rate	17.685	13.424	11.233	10.884	7.393
Cumulative Variance Contribution Rate	17.685	31.109	42.342	53.226	60.619

Note. The item texts presented in this table were translated from the original Chinese version.

**Table 3:** Model fit indices for the confirmatory factor analysis.

Competing Models	χ^2^	df	χ^2^/df	CFI	TLI	RMSEA	SRMR	GFI	AGFI	RMR
Single-Factor Model	2259.116	405	5.578	0.798	0.784	0.095	0.061	0.716	0.674	0.035
Three-Factor Model	1633.846	402	4.064	0.866	0.855	0.078	0.054	0.811	0.781	0.029
Four-Factor Model	1547.729	399	3.879	0.875	0.864	0.076	0.052	0.818	0.788	0.029
Five-Factor Model	1357.421	395	3.437	0.895	0.885	0.070	0.049	0.841	0.812	0.027
Modified Five-Factor Model	1172.556	391	2.999	0.915	0.905	0.063	0.046	0.863	0.838	0.026

**Table 4:** Composite reliability, AVE, Fornell–Larcker matrix, and HTMT values for discriminant validity.

Factor	AVE	CR	1	2	3	4	5
1. Self-Awareness	0.455	0.770	**0.675**	0.206	0.117	0.170	0.175
2. Self-Management	0.557	0.918	0.766	**0.746**	0.137	0.223	0.215
3. Social Awareness	0.448	0.849	0.715	0.745	**0.669**	0.126	0.143
4. Interpersonal communication	0.539	0.853	0.712	0.816	0.818	**0.734**	0.189
5. Sense of Responsibility	0.589	0.896	0.669	0.703	0.807	0.749	**0.768**

Note. Diagonal (bold) = square roots of AVE (Fornell–Larcker criterion). Above diagonal = construct intercorrelations. Below diagonal = HTMT values. All HTMT values are below 0.85, supporting discriminant validity.

**Table 5:** Correlation coefficients between the Social–Emotional Competence Questionnaire and the interpersonal relationship scale.

	M	SD	1	2	3	4	5	6	7
1. Total Score of Interpersonal Relationships	54.02	11.40	1						
2. Total Score of Social–Emotional Competence	122.86	15.08	0.73 **	1					
3. Self-Awareness	12.23	1.75	0.55 **	0.77 **	1				
4. Self-Management	35.85	5.65	0.66 **	0.91 **	0.65 **	1			
5. Social Awareness	29.86	3.35	0.62 **	0.86 **	0.63 **	0.67 *	1		
6. Interpersonal Communication	19.48	3.36	0.70 **	0.89 **	0.64 **	0.77 **	0.71 **	1	
7. Sense of Responsibility	25.42	3.17	0.61 **	0.85 **	0.600 *	0.68 **	0.74 **	0.69 **	1

Note: ** *p* < 0.01. * *p* < 0.05.

**Table 6:** Correlation coefficients between the Social–Emotional Competence Questionnaire and the difficulties in emotion regulation scale.

	M	SD	1	2	3	4	5	6	7
1. Total Score of Difficulties in Emotion Regulation	34.40	14.86	1						
2. Total Score of Social–Emotional Competence	122.86	15.08	−0.41 **	1					
3. Self-Awareness	12.23	1.75	−0.24 **	0.77 **	1				
4. Self-Management	35.85	5.65	−0.43 **	0.91 **	0.65 **	1			
5. Social Awareness	29.86	3.35	−0.32 **	0.86 **	0.63 **	0.67 **	1		
6. Interpersonal Communication	19.48	3.36	−0.34 **	0.89 **	0.64 **	0.77 **	0.71 **	1	
7. Sense of Responsibility	25.42	3.17	−0.35 **	0.85 **	0.60 **	0.68 **	0.74 **	0.69 **	1

Note: ** *p* < 0.01.

**Table 7:** Correlation Coefficients between the Social–Emotional Competence Questionnaire and the Beck Depression Inventory.

	M	SD	1	2	3	4	5	6	7
1. Total Depression Score	16.09	4.33	1						
2. Total Score of Social–Emotional Competence	122.86	15.08	−0.49 **	1					
3. Self-Awareness	12.23	1.75	−0.34 **	0.77 **	1				
4. Self-Management	35.85	5.65	−0.54 **	0.91 **	0.65 **	1			
5. Social Awareness	29.86	3.35	−0.37 **	0.86 **	0.63 **	0.67 **	1		
6. Interpersonal Communication	19.48	3.36	−0.44 **	0.89 **	0.64 **	0.77 **	0.71 **	1	
7. Sense of Responsibility	25.42	3.17	−0.33 **	0.85 **	0.60 **	0.68 **	0.79 **	0.69 **	1

Note: ** *p* < 0.01.

## Data Availability

The datasets generated during and/or analyzed during the current study are available from the first author upon reasonable request.

## Appendix A

The Social–Emotional Competence Questionnaire (SECQ)

Instructions: Please rate the extent to which you agree with each of the following statements on a scale from 1 to 5.

(1 = Strongly Disagree, 2 = Disagree, 3 = Neither Disagree nor Agree, 4 = Agree, 5 = Strongly Agree)

Dimension 1: Self-Awareness

1. I have a clear understanding of my strengths and weaknesses.
2. I know under what circumstances I tend to become angry.
3. I believe that my opinions are worthy of being heard by others.

Dimension 2: Self-Management

4. I can effectively alleviate my own stress.
5. I am able to regulate my behavior.
6. When encountering difficulties, I am confident in my ability to overcome them.
7. I am able to maintain an optimistic mindset over time.
8. I believe that what others can accomplish, I can also achieve through effort.
9. I firmly believe that I can achieve the goals I set for myself.
10. I am able to learn from my successes and accumulate experience.
11. When facing difficulties, I encourage and motivate myself.
12. I believe that once something is planned, it should be followed through to completion.

Dimension 3: Interpersonal communication

13. I am skilled at handling conflicts with others.
14. I am able to resolve misunderstandings with others through communication.
15. When communicating with others, I can clearly express my thoughts and feelings.
16. I am able to initiate conversations with strangers easily.
17. I am able to make friends with different types of people.

Dimension 4: Social Awareness

18. I am able to accept people with different personality traits.
19. I am able to maintain good relationships with my family and friends.
20. I care about others’ feelings.
21. I am able to sense when others are unhappy.
22. When I see others experiencing misfortune, I feel deep sympathy.
23. I receive substantial support from close family members and friends.
24. I have one or more close friends.

Dimension 5: Sense of Responsibility

25. When making choices or decisions, I consider social safety and moral norms.
26. I am able to take responsibility for my actions.
27. I am able to correctly distinguish right from wrong.
28. In dealing with people and situations, I think carefully before acting.
29. The decisions I make are not only responsible to myself but also to others and the collective.
30. I am able to weigh the impact of my decisions on myself, others, and the collective, and make appropriate adjustments when necessary.

(Note: The item texts presented in this Appendix A were translated from the original Chinese version.)
